# The Treatment of Measles

**Published:** 1884-02

**Authors:** 


### The Treatment of Measles.

Dr. D. Maclean, of Glasgow, in the *Lancet*, calls attention to a method of treating measles, which has been found, in his hands, to meet with what he calls universal success.

As this disease is considered one of the zymotic class, two principal things must be considered in its treatment: (1.) The management of the ferment, or whatever it is; and (2.) the management of the effects of this ferment upon the system. The most marked of these latter present themselves in the effects of the ailment upon the mucous membrane of the lungs, and it is from its action there we have the immediate cause of the ensuing death, or the prolonged ill-health afterwards. The line of action to follow is: (1.) To relieve the congestion of the mucous membrane, which is the immediate cause of danger; and (2.) to destroy or reduce the violence of the disease itself. This, Dr. Maclean is in the habit of doing, he believes successfully, by giving (say to a child of two or three years of age) a teaspoonful, in water, of the following mixture, every three hours: ipecacuanha wine, half a drachm; syrup of squills, half an ounce; quinine, two grains; acetate of ammonia solution, to two ounces. The quinine is increased according to age. It may be necessary to add to, or modify, the form in which this plan of treatment is carried out, as when the irritation or cough is persistently great, then the addition of a little tincture of hyoscyamus is all that is necessary. If the stomach is irritable, it may be necessary to omit the quinine from the mixture. But as it is essential that it be introduced into the system for the destruction of the ferment, it can be administered separately by giving it in powder, mixed with saccharated carbonate of iron, which diminishes the irritant action of the quinine that takes place when the drug is given alone.

This form of treatment for measles he regards good in all types of the disease, whether the attack be mild or severe, and more especially valuable when we have that dangerous form, in which the eruption is of a deep-purplish color, a form which is generally recognized as being the most fatal.—*Md. Med. Jour.*

